# Supplementary material for: Genome-Wide Methylation Profiling in Canine Mammary Tumor Reveals miRNA Candidates Associated with Human Breast Cancer
Source: Cancers (Basel). 2019 Sep 29;11(10):1466. doi: 10.3390/cancers11101466 (PMC6827104; doi:10.3390/cancers11101466)
Supplement: Supplementary file 1 [file cancers-11-01466-s001.zip › cancers-542893-suppl-final/cancers-542893-supplementary-final.docx]

**Supplementary materials**


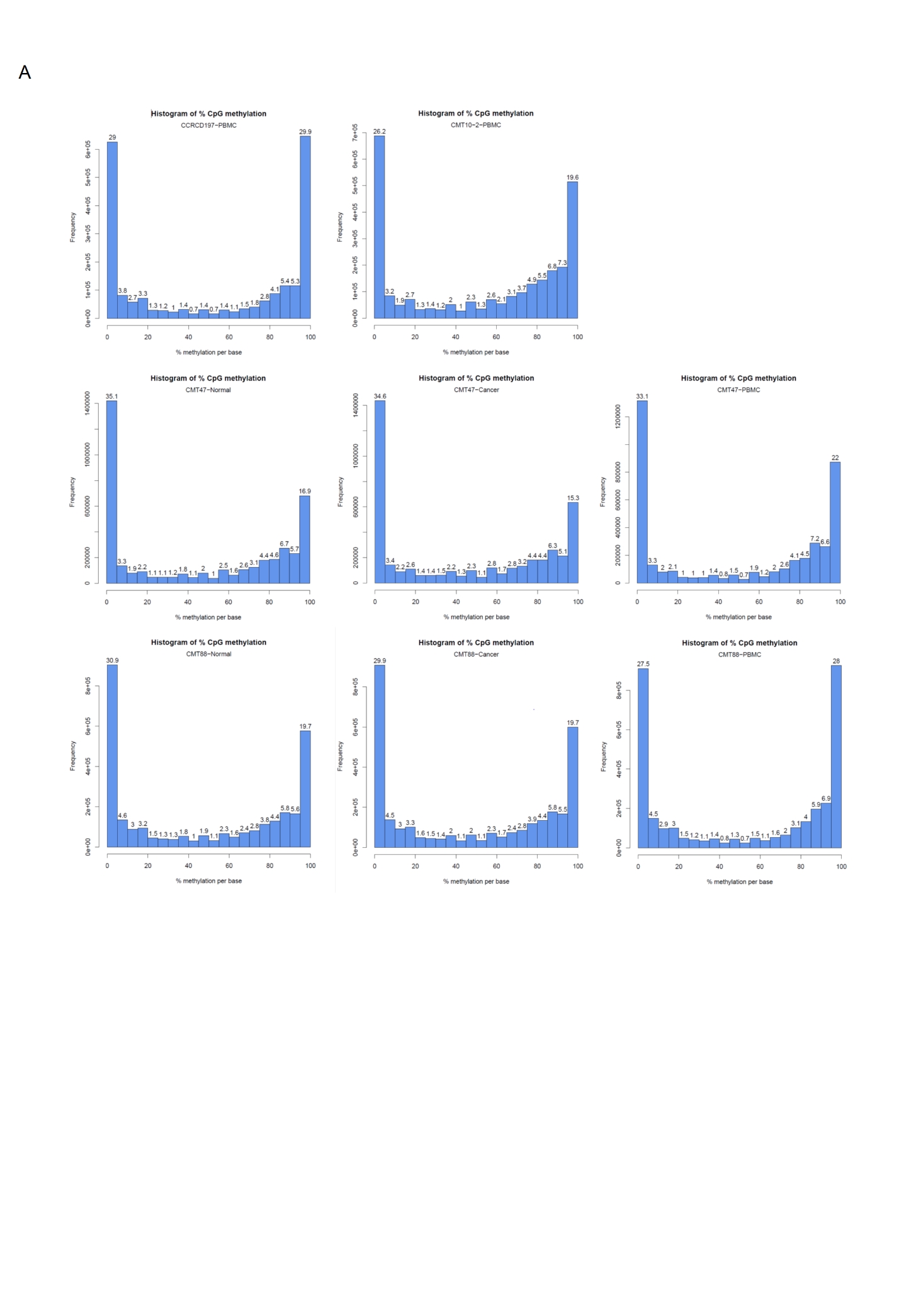


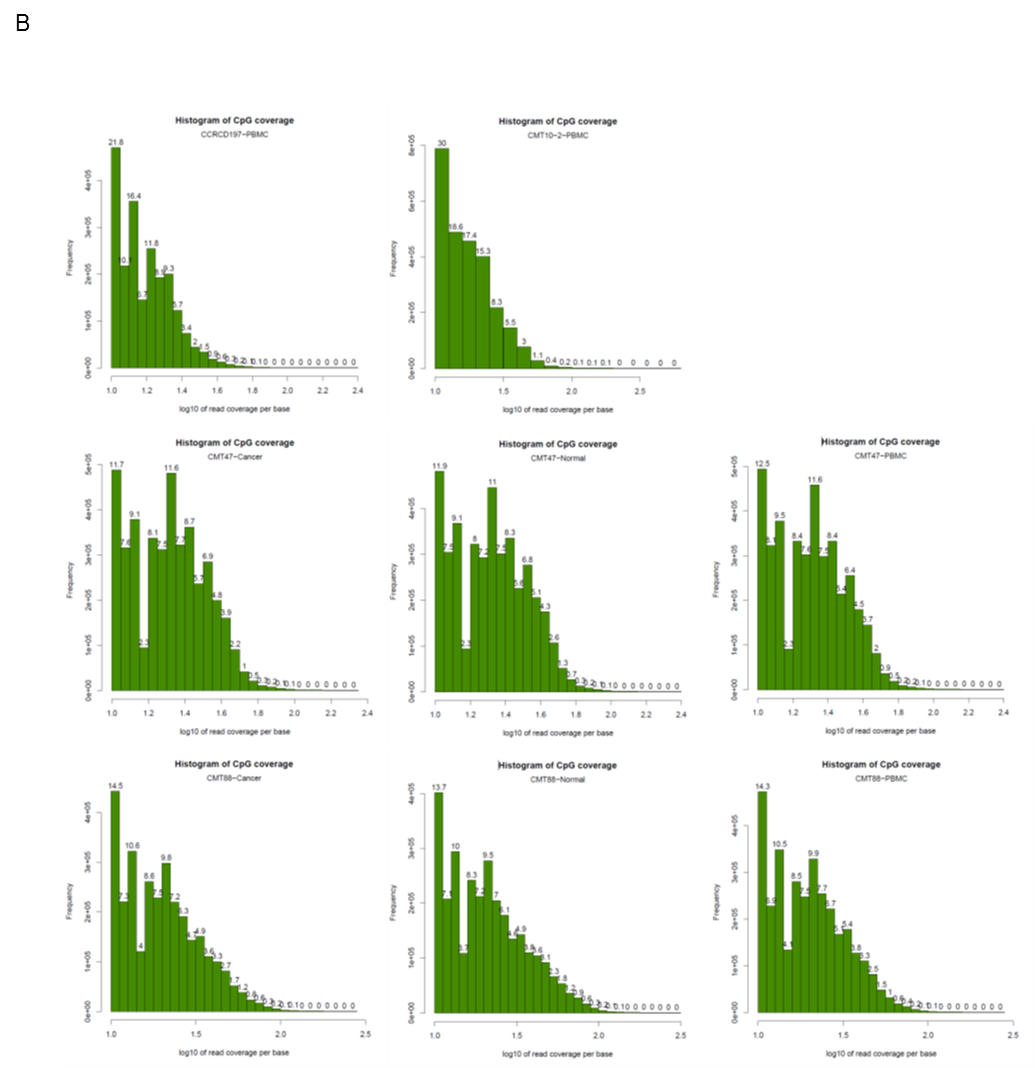


**Figure S1.** Sequencing QC. (**A**) Histogram of methylation distribution of RRBS samples. X-coordinate represented methylation percentage (%) and y-coordinate represented read frequency. (B) The frequency of methylation at any CpG region has very similar patterns across samples.


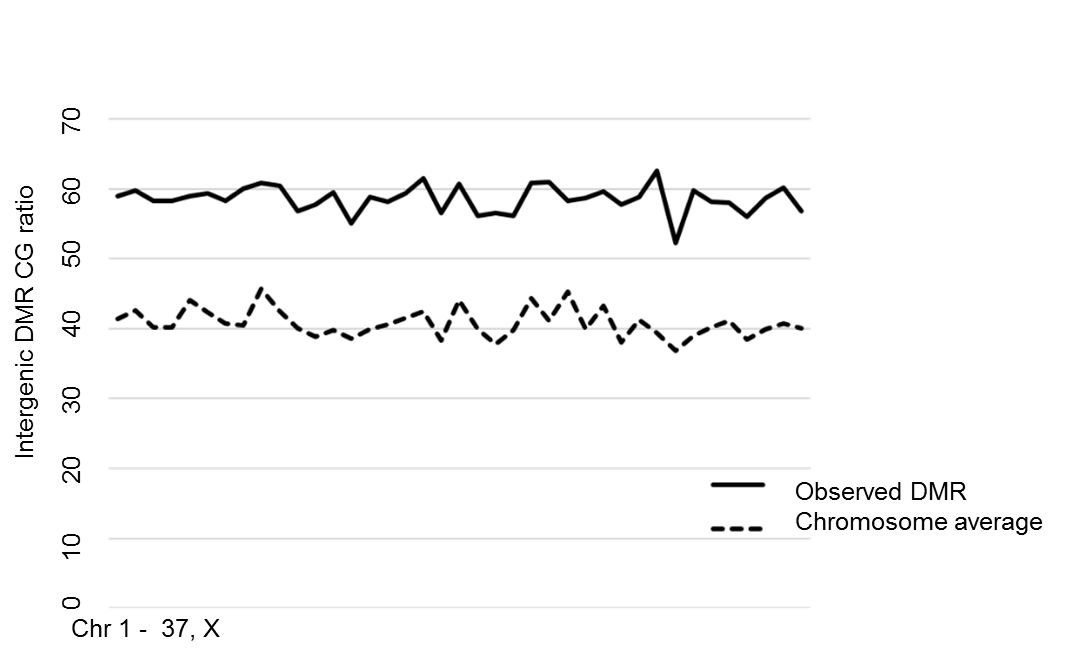


**Figure S2.** Intergenic DMRs CpG CG ratio**.** X-coordinate represented chromosome number and y-coordinate represented CG ratio. Full line was assayed intergenic DMRs CG ratio, dotted line was chromosome average CG ratio.


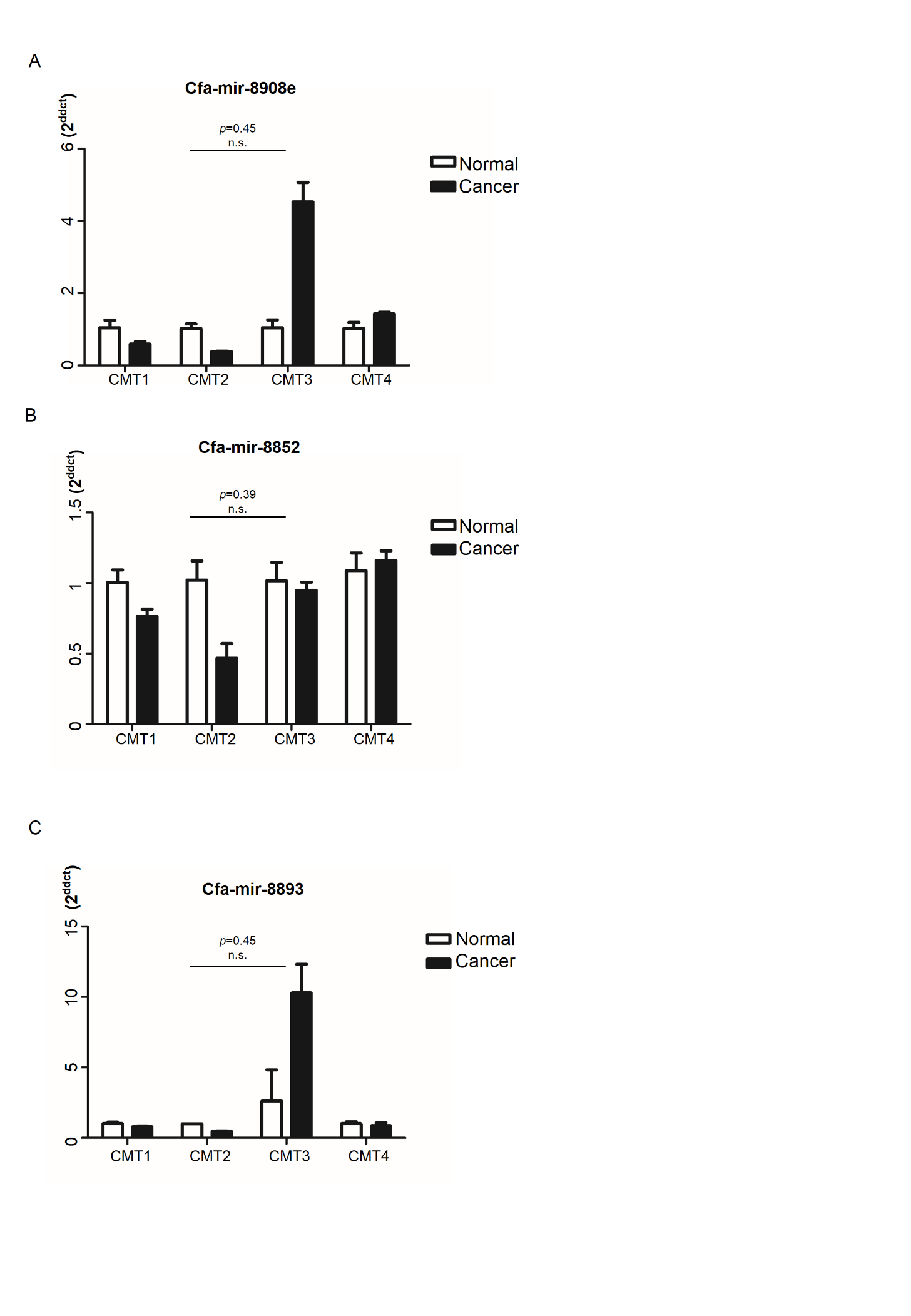


**Figure S3.** Canine novel miRNAs expression level in CMT. (**A**–**C**) cfa-mir-8908e, 8852 and 8893 were not significantly dysregulated. Data are expressed as means ± SD. n.s.: not significant.


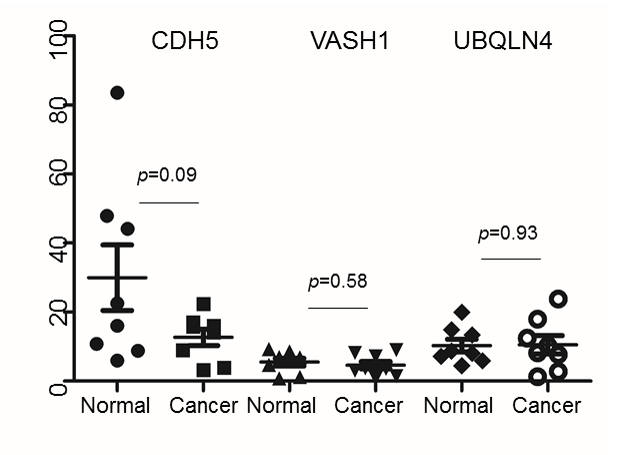


**Figure S4.** Canine novel miRNA targeted gene expression level in CMT. Among Cfa-mir-8832 targeted top 5genes, three genes were not significantly downregulated.


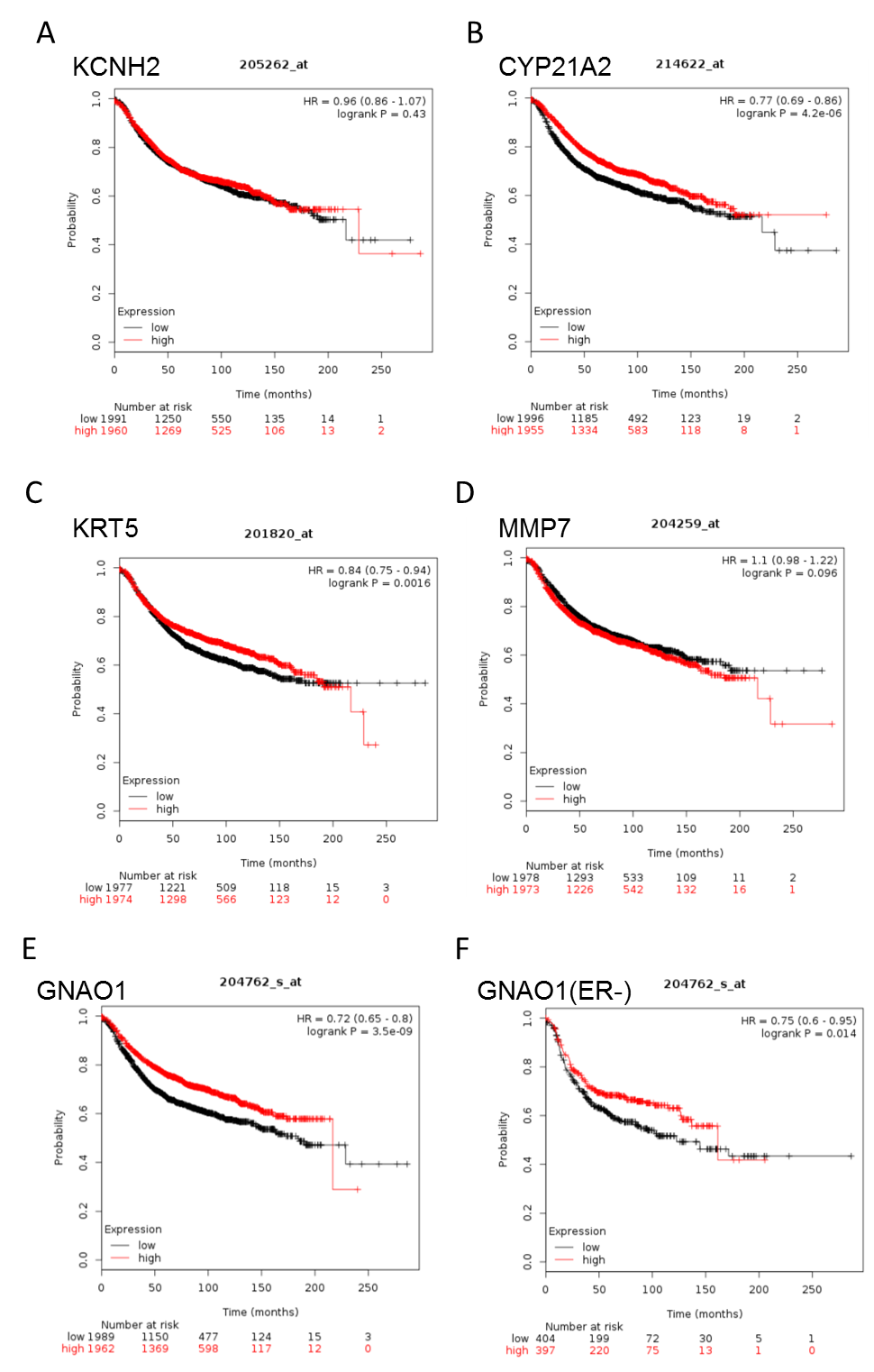


GNAO1

GNAO1(ER-)

E

F


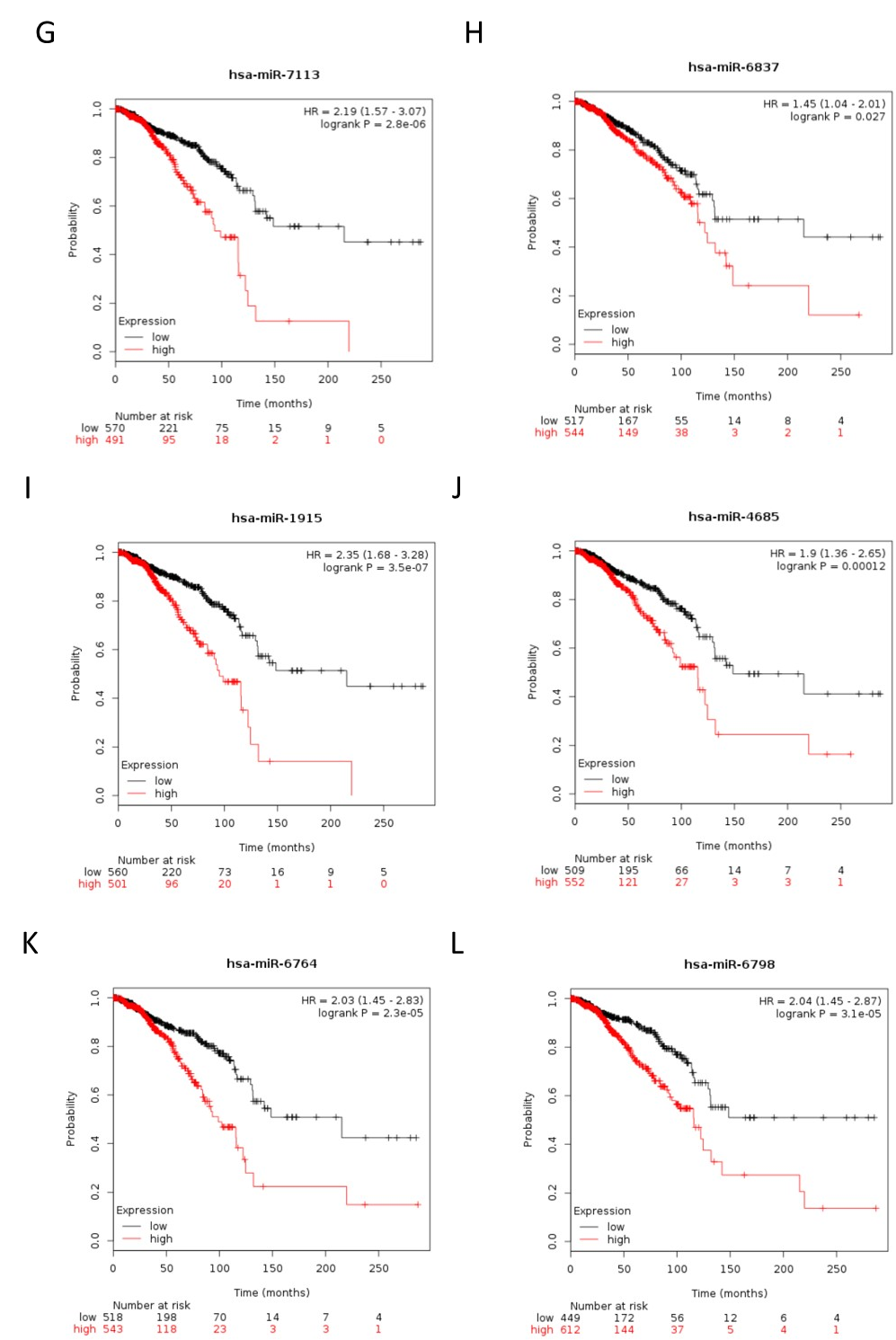


**Figure S5.** Kaplan-Meier plots of identified target genes and miRNAs. (**A**–**F**) Identified target genes are associated with breast cancer patient survival. (**G**–**L**) Identified orthologous miRNAs are associated with breast cancer patient survival.


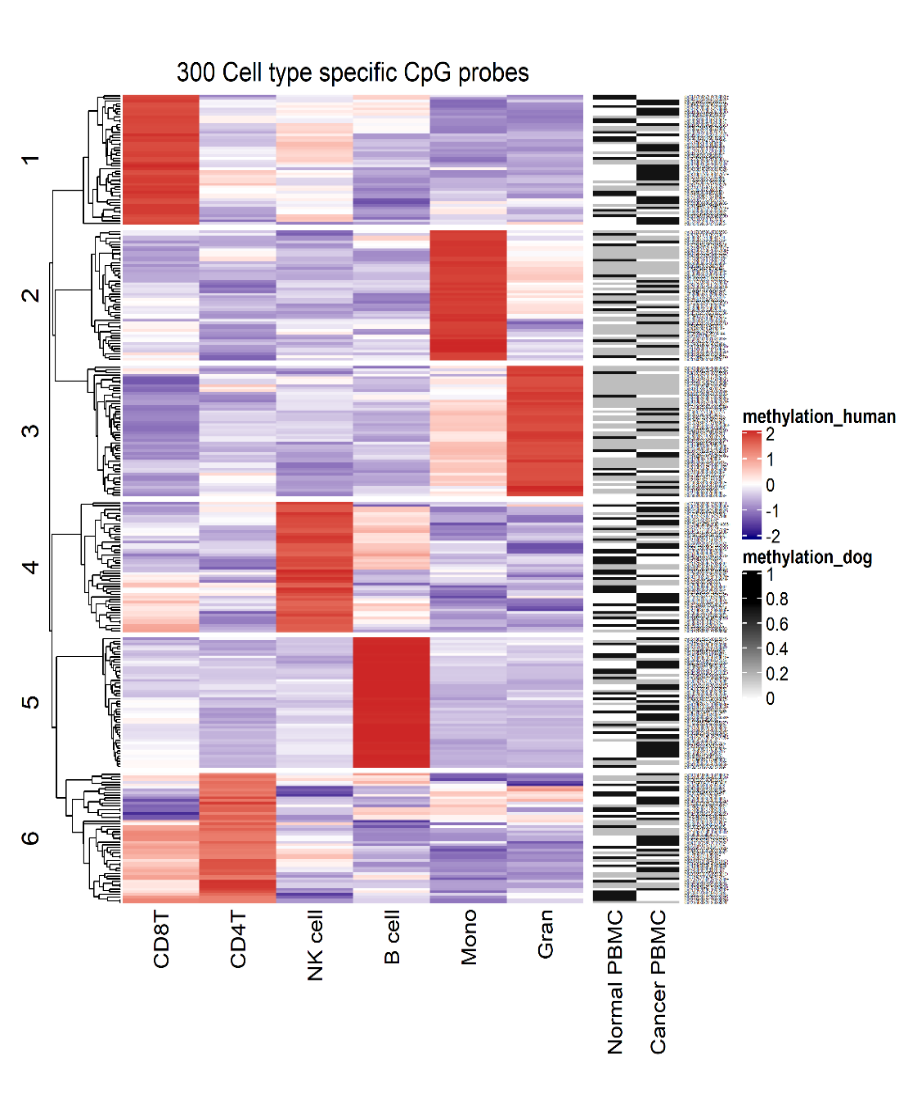

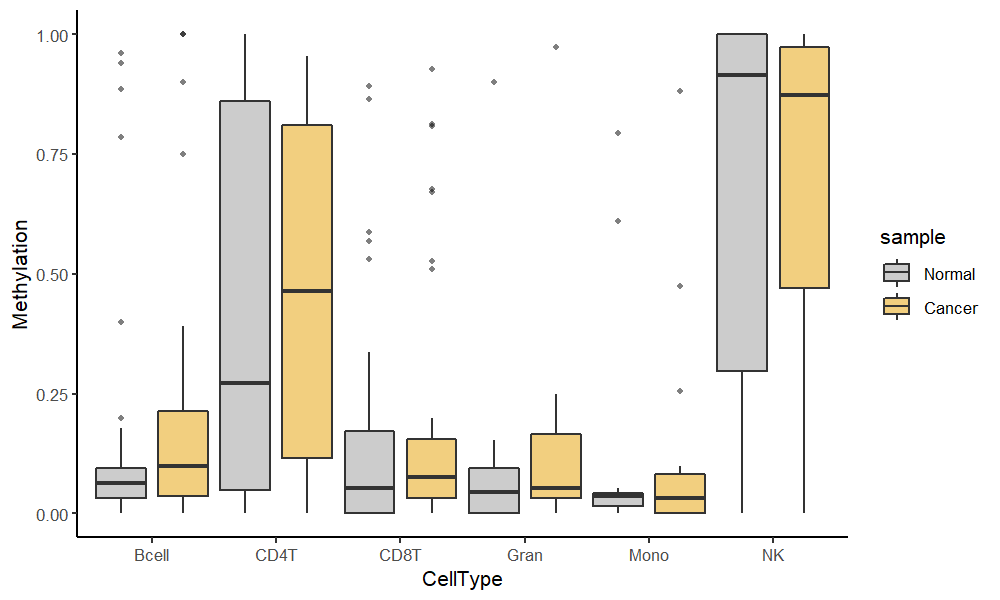


**B**

**A**

**Figure S6.** Estimation of cellular population in normal and cancer PBMCs using cell-type specific methylated CpG sites. (**A**) 300 cell type-specific methylated CpG sites were selected from previous human PBMC methylome study to estimate cellular composition changes in cancer PBMC [52]. Heatmap clustered by Euclidean distance shows each CpG methylation (“Red” means high methylation level) is prominent in one cell type (50 CpGs are selected for each cell type). Then, 300 CpG sites were liftovered to dog genome using UCSC liftOver and methylation levels for each probe are calculated in normal and mammary gland cancer PBMC (“Black” represent higher methylation level). (**B**) Boxplot shows methylation differences between normal (gray) and dog mammary gland cancer (yellow) PBMCs in cell type specific CpG sites. Methylation changes of cell type specific probes could indirectly explain the alteration of cellular compositions in cancer PBMCs.
